# Aspirin and Preeclampsia Prevention in Patients With Abnormal Uterine Artery Blood Flow

**DOI:** 10.5812/ircmj.17175

**Published:** 2014-08-05

**Authors:** Hamidreza Talari, Elahe Mesdaghinia, Masoumeh Abedzadeh Kalahroudi

**Affiliations:** 1 Department of Radiology, Kashan University of Medical Sciences, Kashan, IR Iran; 2 Anatomy Research Center, Kashan University of Medical Sciences, Kashan, IR Iran; 3 Trauma Research Center, Kashan University of Medical Sciences, Kashan, IR Iran; 4 Department of Obstetrics Gynecology, Kashan University of Medical Sciences, Kashan, IR Iran; 5 Trauma Nursing Research Center, Kashan University of Medical Sciences, Kashan, IR Iran; 6 Department of Midwifery, Kashan University of Medical Sciences, Kashan, IR Iran

**Keywords:** Pre-Eclampsia, Blood Flow, Prevention, ASA

## Abstract

**Background:**

Preeclampsia is one of the leading causes of maternal mortality and morbidity. Its prevalence varies between 10-25% among high-risk pregnant patients.

**Objectives:**

The aim of this study was to determine whether treatment with acetylsalicylic acid (ASA) reduces the incidence of preeclampsia among pregnant women with abnormal uterine artery flow.

**Patients and Methods:**

In this double-blind, placebo controlled trial, 80 high-risk pregnant women with preeclampsia, who had abnormal findings on Doppler ultrasonography at 12-16 weeks of pregnancy (unilateral notch with RI ≥ 0.65 or bilateral notch with RI ≥ 0.55), were randomly divided into two groups; the intervention group was treated with ASA tablet 80 mg, one tablet per day, and the control group was given placebo. Then patients were followed until the end of their pregnancy period, and pregnancy outcomes, including development of preeclampsia, the intrauterine growth retardation (IUGR), prematurity, type of delivery, birth weight, and Apgar score at one and five minutes were assessed. Data were analyzed using the student's t-test, chi-square or Fisher's exact test, and multivariate logistic regression. P values less than 0.05 were considered statistically significant.

**Results:**

There were no significant differences between the two groups in terms of baseline characteristics. There was a significant difference between the ASA and placebo groups in the incidence of preeclampsia (2.5% versus 22.5%), adjusting for the neonatal and maternal covariates.

**Conclusions:**

ASA prophylaxis can be used for prevention of preeclampsia in high-risk patients with abnormal uterine artery.

## 1. Background

Preeclampsia is a disorder characterized by a widespread vascular endothelial malfunction and vasospasm and the leading cause of maternal mortality and morbidity (1). Although the incidence of this phenomenon is low, there is a potential for adverse serious maternal outcomes including the HELLP syndrome (hemolysis elevated liver enzymes low platelets counts), coagulopathy, eclampsia, and stroke, as well as adverse neonatal complications due to intrauterine growth restriction (2, 3). The prevalence of preeclampsia is about 5-10% in general pregnant population (4) and varies between 10-25% among high-risk pregnant patients (4, 5). According to the recent reports of the World Health Organization, this even leads to over 100000 deaths each year worldwide, especially in developing countries (6). hence, accurate prediction of preeclampsia is paramount to providing appropriate antenatal surveillance and therapy, in an effort to improve perinatal outcomes (7). In this context, the assessment of uteroplacental circulation is the potential target. This parameter is usually assessed by means of Doppler ultrasonography of the uterine arteries (8-13). In fact, preeclampsia is felt to be the result of abnormal placenta formation, involving abnormal trophoblast invasion of spiral arteries and an increase in vascular resistance in the uteroplacental circulation (14-17). Therefore, alterations observed in uterine artery Doppler ultrasound images can be the main key to predict adverse perinatal outcomes in clinical conditions. There are no approved methods for identification of vasospasm, but Doppler ultrasonography is an invasive methods and appropriate test for prediction of vascular involvements (18). Its sensitivity and specificity for prediction of preeclampsia are 43% and 67%, respectively (19). Although in high-risk patients this method has 81.4% sensitivity (20), abnormal finding in the Doppler sonography images include the presence of unilateral notch with RI ≥ 0.65 or presence of bilateral notch with RI ≥ 0.55 (20, 21).

Currently, there is no effective drug for treatment of preeclampsia; therefore, its prevention is very important. Low dose acetylsalicylic acid (ASA) is a drug that decreases thromboxane production without reducing the prostacyclin production (22), preventing vasoconstriction and coagulation problems which are the characteristic of preeclampsia (23). Administration of ASA is safe for both mother and fetus (24-26). The proposed ASA dosage varies between 60-150 mg per day and has been initiated between 13 to 26 weeks of pregnancy in different studies (22, 27, 28). One study showed that taking 150 mg ASA daily after 23 week of gestation in patients with abnormal Doppler indices, did not stop progression of preeclampsia (28). Conversely, another study showed that ASA treatment resulted in a significant decrease of preeclampsia in patients with abnormal Doppler findings of uterine arteries (29). In 2000, Harrington et al. published a paper in which the use of ASA in high risk patients, based on Doppler finding on the 20th week of gestation, did not make a significant difference on the incidence of preeclampsia, suggesting that future studies should aim on assessment of ASA usage early in pregnancy (30). Some studies have shown that ASA has decreased the risk of preeclampsia by 10-20%, considering this as a low to intermediate risk reduction. Therefore, only high risk women benefit from ASA (31-33). A study concluded that low-dose aspirin starting at 14-16 weeks in high-risk women with abnormal Doppler of uterine arteries declined the risk of sever preeclampsia (18).

## 2. Objectives

Regarding controversial results in different studies, in addition to the importance of preeclampsia prevention and lack of any trials in our country, we conducted a double-blind, placebo-controlled study to determine whether treatment with ASA can reduce the incidence and complications of preeclampsia among pregnant women with abnormal uterine artery Doppler ultrasound images.

## 3. Patients and Methods

Eighty pregnant women attending Shabihkhani Antenatal Clinic in Kashan, Iran, were enrolled in a randomized double-blinded controlled trial. Criteria for inclusion were presence of a high-risk factor for preeclampsia, such as previous history of the disease, essential hypertension, positive family history or underlying vascular disorder, gestational diabetes mellitus, or maternal age < 20 years or > 40 years. Subjects with a known history of salicylate allergy or present or past peptic ulcer, as well as cases with other medical disorders such as chronic renal disorders, thyroid diseases, and hepatic and cardiac disorders were excluded from the study.

In addition to performing the routine ultrasound in all the participants to evaluate pregnancy at the time of booking, all the patients underwent Doppler ultrasonography of the uterine artery through either transabdominal or transvaginal route at 12-16 weeks of pregnancy. The outcome measures were presence of the uterine artery diastolic notch (bilateral or unilateral), and the resistance index (RI). Based on these indices, abnormal finding in the Doppler sonography image was defined as presence of unilateral notch with RI ≥ 0.65 or bilateral notch with RI ≥ 0.55 (20, 21).

**Figure 1.**
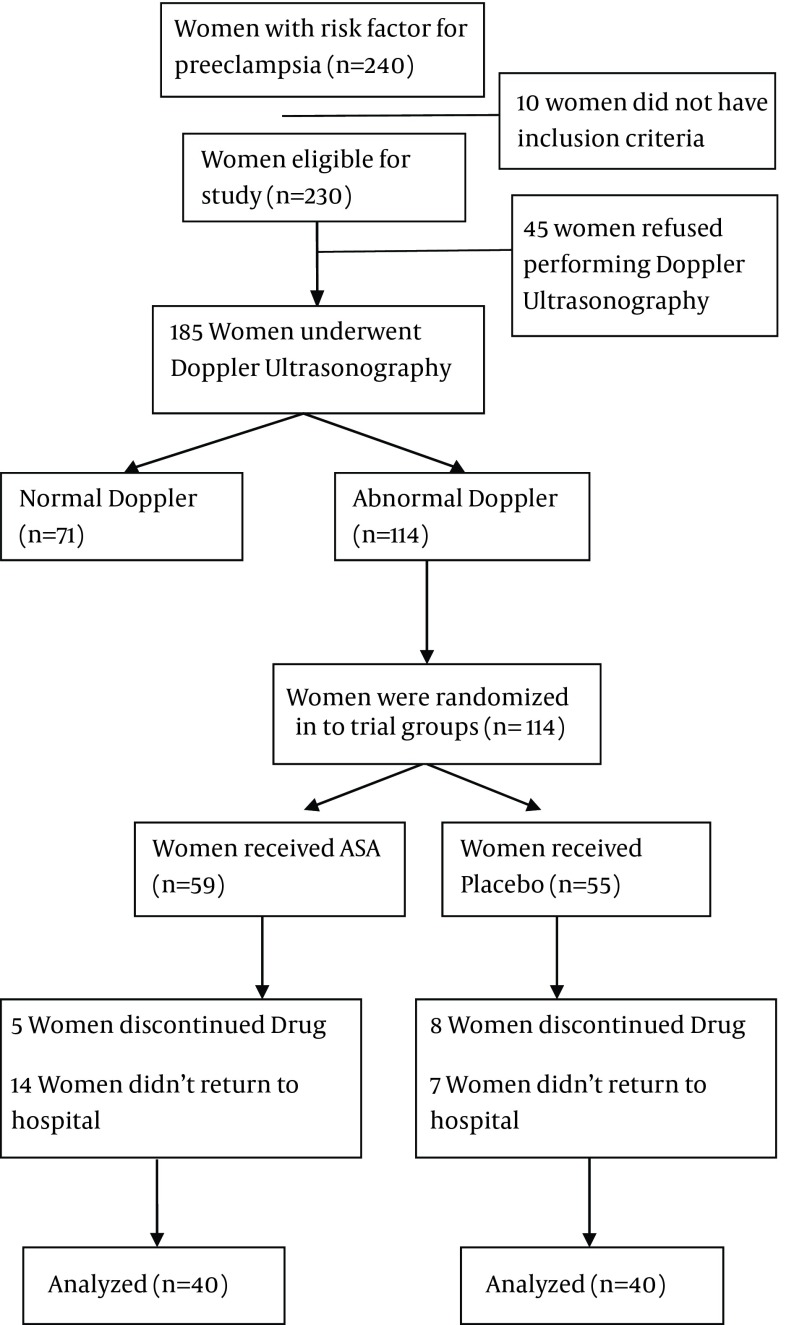
Study Design

Patients shown to have normal Doppler findings were not included in the study. The included ones agreed to sign consents and were randomly divided into two groups: group 1 including patients who were referred on odd days of the week (40 patients), treated with ASA 80 mg one tablet per day after lunch, and the control group including patients who were referred on even days of the week (40 patients), given placebo through the same routine. The drug prescription and allocation key were kept by one author who did not have any role in patients’ follow ups or assessing the outcomes. Figure 1 is a flowchart of the study design.

All the women were examined throughout pregnancy and after that regularly for pregnancy outcome assessment. The main outcome criteria were the development of preeclampsia, the intrauterine growth retardation (IUGR), prematurity, type of delivery, birth weight, and Apgar scores at one and five minutes if less than 5. The neonatal birth weight was documented for those with IUGR. Preeclampsia was defined as development of hypertension (140/90 mmHg or more) plus proteinuria (> 300 mg protein in the 24-hour urine sample) (4).

Results were reported as mean ± standard deviation (SD) for quantitative variables and percentages for categorical variables. The groups were compared using the student's t-test for continuous variables and chi-square test (or Fisher's exact test if required) for categorical variables. Predictors exhibiting a statistically significant relation with the occurrence of preeclampsia in the two groups were used for multivariate logistic regression analysis to investigate their independence as predictors. Odds ratio (OR) and 95% confidence interval (CI) were calculated. This study was performed with the power of 80%. P values of 0.05 or less were considered statistically significant. All the statistical analyses were performed using SPSS version 13.0 for windows (SPSS Inc., Chicago, IL, USA). This study was approved by the Ethical Committee of Kashan University of Medical Sciences and registered in Iranian Registry of Clinical Trials with IRCT NO. 138809232854N1.

## 4. Results

There were no significant differences in the baseline characteristics including demographics, clinical data, as well as medical history, between the two groups (Table 1). The two groups were also similar in terms of history of preeclampsia, diabetes mellitus, and hypertension. Totally, 10 (12.5%) of the study population developed preeclampsia. There was a significant difference between the ASA and placebo groups in the incidence of preeclampsia (2.5% versus 22.5%). Comparison between ASA and control groups with regard to the incidence of preeclampsia adjusting for neonatal and maternal covariates (Table 2) showed a trend towards a lower risk of preeclampsia in the ASA group. In fact, ASA reduced the risk of preeclampsia approximately 11 times. The multivariable regression model also showed that being a male neonate and multiparity could also effectively prevent the incidence of preeclampsia. None of the cases experienced IUGR, prematurity, or Apgar scores at one and five minutes of less than 5.

**Table 1.** Baseline Characteristics of the Study Population (n = 80) ^a^

Characteristics	Intervention Group (n = 40)	Control Group (n = 40)	P Value ^b^
**Maternal age, y**	27.8 ± 4.5	27.0 ± 5.9	0.490
**Maternal age group**			0.224
< 20	4 (10.0)	5 (12.5)	
20-40	26 (65.0)	22 (55.0)	
> 40	10 (25.0)	13 (32.5)	
**Parity**	2.2 ± 0.9	2.0 ± 1.1	0.343
**Parity**			0.260
1	10 (25.0)	20 (50.0)	
2	15 (37.5)	7 (17.5)	
≥ 3	15 (37.5)	13 (32.5)	
**Body mass index, kg/m** ^**2**^	25.1 ± 3.2	25.5 ± 3.8	0.251
**Nationality **			0.057
Iranian	39 (97.5)	33 (82.5)	
Afghan	1 (2.5)	7 (17.5)	
**Type of delivery**			0.262
Vaginal	24 (60.0)	19 (47.5)	
Cesarean	16 (40.0)	21 (52.5)	
**Gestational age, w**	39.2 ± 0.7	39.1 ± 0.8	0.758
**Medical history**			
Preeclampsia	9 (22.5)	9 (22.5)	0.999
Hypertension	12 (30.0)	12 (30.0)	0.999
Diabetes mellitus	1 (2.5)	4 (10.0)	0.350
**Gender of newborn**			0.654
Female	20 (50.0)	22 (55.0)	
Male	20 (50.0)	18 (45.0)	
**Weight of neonate, kg**	3.2/0.3	3.2/0.5	0.650

^a^ Data are presented as Mean ± SD.

^b^ P < 0.05 was considered significant.

**Table 2.** Comparison Between Aspirin and Control Groups With Regard to the Incidence of Preeclampsia, Adjusting for Neonatal and Maternal Covariates

Characteristics	Odds Ratio	95% Confidence Interval	Multivariable P Value ^a^
**Aspirin usage**	11.323	1.360-94.248	0.007
**History of preeclampsia**	1.185	0.228-6.152	0.999
**History of hypertension**	0.600	0.153-2.355	0.477
**History of diabetes**	0.929	0.870-0.991	0.999
**Female neonate**	10.091	1.213-83.952	0.016
**Primiparity**	19.636	2.341-164.696	0.001

^a^ P < 0.05 was considered significant.

## 5. Discussion

There are a few evidences recommending ASA prophylaxis as a primary preventive measure in women at high risk of developing preeclampsia. According to recent findings, low-dose ASA prophylaxis can be considered in women at high risk for preeclampsia. In the present study on pregnant women with abnormal uterine artery flow, by commencing treatment of ASA for these patients, we could reduce the incidence of preeclampsia compared with the placebo group. In fact, we showed that the incidence of preeclampsia was lower in the ASA group than in the control group. These findings were consistent with some recent studies regarding ASA therapy for prevention of preeclampsia, following uterine artery Doppler studies, which showed tendency towards benefit (29). In a study on 86 high-risk women with abnormal Doppler findings in 12-14 weeks of pregnancy, aspirin treatment resulted in lowering the risk of preeclampsia from 37.2% in the placebo group comparing to 11.6% in the aspirin group (34). In another study by Bower et al. on women with abnormal uterine artery flow velocity waveforms, there was 29% incidence of preeclampsia in the ASA group and 41% in the placebo group, showing a significant difference (25).

However, the results of Harrington et al. study on 216 high-risk women at 17-23 weeks of gestation showed that aspirin could not prevent preeclampsia (30). Furthermore, Yu’s et al. study showed that use of aspirin after 23 weeks of gestation in women with abnormal Doppler finding could not prevent the progression of preeclampsia (28). These findings were not consistent with our study, because in these studies performing ultrasonography and thereby starting aspirin treatment occurred in the second trimester, which was later than the gestational age at which we started the ASA treatment. It probably can explain why aspirin was not effective in their studies.

Logistic regression analysis showed that nuliparity was a risk factor of preeclampsia, which was inconsistence with the studies reflected in reference books (4). The fetus gender is also a predictive factor for preeclampsia. It is along with Shiozaki et al. study results, identifying that female fetuses were at a higher risk of preeclampsia (35). Regarding the safety of ASA in pregnancy and its positive effects on prevention of preeclampsia, we recommend the use of this drug in high-risk patients, especially nulliparous mothers with female fetuses. In addition, we emphasize on the importance of preeclampsia prevention in high-risk patients and prophylaxis therapy with ASA for reducing its incidence.

According to our findings and consistent with previous trials, ASA prophylaxis at the first trimester of pregnancy in high-risk patients with abnormal uterine artery flow, can be used for prevention of preeclampsia. The importance of this study was magnified through its very low costs, the relative safety, and the wide availability of ASA throughout the world.
